# 

**Published:** 1843-01

**Authors:** 


### CONTENTS OF No. XXIX.

OF THE

### British and Foreign Medical Review.

JANUARY, 1843.

---

### Part First.—Analytical and Critical Reviews.

PAGE

Art. I.—1. Statistical Reports on the Sickness, Mortality, and Invaliding among Her Majesty's Troops serving in Ceylon, the Tenasserim Provinces, and the Burmese Empire . . . . . . . 1

2. Statistical Reports on the Health of the Navy for the years 1830, 1831, 1832, 1833, 1834, 1835, and 1836. South American, West Indian, and North American, Mediterranean, and Peninsular Commands . . . . ib.

3. Statistical Reports on the Health of the Navy, for the years 1830, 1831, 1832, 1833, 1834, 1835, and 1836. Part II. (Cape of Good Hope and West Coast of Africa, and East India Commands, Home, and various forces) . ib.

Art. II.—Erinnerungen an England. 1841. Von Dr. Marx . . 19

Recollections of England. 1841. By Dr. K. F. H. Marx.

Art. III.—Clinique Chirurgicale de l'Hôpital de la Pitié. Par J. Lisfranc . 30

Clinical Surgery of the Hospital of La Pitié. By J. Lisfranc.

Art. IV.—Notes on the United States of North America, during a Phrenological Visit in 1838-9-40. By G. Combe . . . . . 52

Art. V.—1. Das Erbrechen; die Wirkung und Anwendung der Brechmittel. Von J. W. Arnold . . . . . . 60
Vomiting: the Action and Use of Emetics. By Dr. Arnold.

2. Die Lehre vom Erbrechen; Nach Erfahrungen und Versuchen. Von Dr. Julius Budge, &c. Mit einer Vorrede von Dr. Friedrich Nasse . ib.
The Doctrine of Vomiting, from Observations and Experiments by Dr. Budge. With a Preface, by Dr. Nasse.

Art. VI.—Third Annual Report of the Registrar-General of Births, Deaths, and Marriages in England . . . . . . . 74

Art. VII.—1. Handboek der Ziektekundige Ontleedkunde. Door W. Vrolik . 83

Manual of Morbid Anatomy. By W. Vrolik, m.d.

2. Specielle pathologische Anatomie. Von Dr. Karl Ewald Hasse . . ib.
Special Pathological Anatomy. By Dr. K. E. Hasse.

3. Handbuch der Pathologischen Anatomie. Von Carl Rokitansky . . ib.
Manual of Morbid Anatomy. By Carl Rokitansky, m.d.

Art. VIII.—De Herophili Celeberrimi Medici Vita, Scriptis, atque in Medicina Meritis. Auctore Car. Frid. Henr. Marx . . . 106

Art. IX.—A Practical Treatise on the Diseases of the Scalp. By J. E. Erichsen 114

4292

PAGE

ART. X.—1. Fallacies of the Faculty; with the Principles of the Chrono-thermal System. In a Series of Lectures. By SAMUEL DICKSON, M.D. . . 124

2. Erreurs des Médecins, ou Systême Chrono-thermal; traduit de l'Anglais du Dr. Dickson. Par MALVIUS, A.D.C. . . . . ib.

ART. XI.—Recherches sur les Abcès Multiples, et sur les Accidents qu'amène la présence du Pus dans la Systême Vasculaire. Thèse par F. D'ARCET, M.D. 131

Researches on Multiple Abscesses, and on the Morbid Effects of the presence of Pus in the Vascular System. By F. D'ARCET, M.D.

ART. XII.—1. Essays on the Philosophy of Vitality, as contradistinguished from Chemical and Mechanical Philosophy, and on the modus operandi of Remedial Agents. By MARTYN PAINE, A.M. M.D. . . . . 134

2. The Cyclopædia of Anatomy and Physiology. Edited by R. B. TODD, M.D. F.R.S. —Article "Life," by W. B. CARPENTER, M.D. . . . ib.

ART. XIII.—Medico-Chirurgical Transactions. Vol. XXV. . . 147

1. A case of cyanosis, depending upon transposition of the aorta and pulmonary artery, by Dr. WALSHE . . . . . ib.
2. Case of aneurism of the ascending aorta, by Mr. BECK . . . 148
3. On the structure and functions of the human placenta, by J. DALRYMPLE, Esq. ib.
4. Relation between the symmetry and diseases of the body, by J. PAGET . 149
5. Case of extensive disease of the pancreas, by J. A. WILSON, M.D. . 151
6. Remarks on typhus fever, by J. BOSTOCK, M.D. . . . . . ib.
7. Laryngitis relieved by operation, by JOHN WILSON, M.D. . . ib.
8. Illness and death of an entire family, by the same author . . ib.
9. Case of congenital cataract, by Mr. STAFFORD . . . 152
10. On diseases which affect corresponding parts of the body, by W. BUDD, M.D. 149
11. On cases of plague, by M. PEZZONI . . . . . . 152
12. Tubercle of the brain in children, by P. HENNIS GREEN, M.B. . . ib.
13. Case narrated by Mr. DUNN . . . . . . 153
14. Stricture of the trachea, by Mr. W. C. WORTHINGTON . . ib.
15. Tumours in the head and face, by Mr. ANCELL . . . ib.
16. Malformation of the heart, by THEOPHILUS THOMPSON, M.D. . . 154
17. Petechial cowpox, by Dr. GREGORY . . . . . . ib.
18. Ulceration of the duodenum after burns, by Mr. CURLING . . 155
19. Malformation of the heart, by Dr. T. B. E. FLETCHER . . . ib.
20. Tumours in the neck, by B. PHILLIPS . . . . . ib.

On a variety of False Aneurism. By ROBERT LISTON, F.R.S. &c. &c. (with two woodcuts) . . . . . . . . 155

ART. XIV.—On Injuries of the Head affecting the Brain. By G. J. GUTHRIE, F.R.S. 161

ART. XV.—The Natural History of Man; comprising Inquiries into the Modifying Influence of Physical and Moral Agencies on the different Tribes of the Human Family. By JAMES COWLES PRICHARD, M.D. F.R.S. M.R.I.A. &c. . 180

ART. XVI.—The Anatomy of Sleep, or the Art of procuring sound and refreshing Slumber at will. By EDWARD BINNS, M.D. &c. . . . . 184

ART. XVII.—A System of Practical Surgery. By W. FERGUSSON, F.R.S.E. With 246 Illustrations by BAGG . . . . . . . 188

ART. XVIII.—Descriptive and Illustrated Catalogue of the Physiological Series of Comparative Anatomy, contained in the Museum of the Royal College of Surgeons in London. Four vols. 4to. . . . . . . 195

ART. XIX.—Fourth Report of the Resident-Physician of the County of Middlesex Pauper Lunatic Asylum at Hanwell, October 1st, 1842 . . . 218

ART. XX.—The Physical Diagnosis of Diseases of the Lungs. By WALTER HAYLE WALSHE, M.D. . . . . . . . 223

### PART SECOND.—Bibliographical Notices.

PAGE

ART. I.—Ausführliche Encyklopädie der gesammten Staatsarzneikunde. Von GEORG FRIEDERICH MOST . . . . . . 225

A Complete Encyclopædia of State Medicine. By Dr. GEO. FRED. MOST.

ART. II.—Observations on Ulcers of the Legs and other parts, showing that the most obstinate and intractable cases may be speedily cured by mild methods of treatment, with Remarks on Scrofulous Disorders. By A. MAXFIELD . 226

ART. III.—Clinical Midwifery; with the Histories of Four Hundred Cases of Difficult Labour. By ROBERT LEE, M.D. F.R.S. . . . . ib.

ART. IV.—The Prescriber's Pharmacopœia: containing all the Medicines in the London Pharmacopœia, arranged in classes according to their action, with their composition and doses. By a PRACTISING PHYSICIAN. Second Edition, corrected and enlarged . . . . . . 227

ART. V.—Recherches Anatomiques, Physiologiques, Pathologiques, et Séméiologiques, sur les Glandes Labiales. Par A. A. SEBASTIAN . . ib.

Anatomical, Physiological, Pathological, and Semeiological Researches on the Labial Glands. By A. A. SEBASTIAN.

ART. VI.—The Retrospective Address delivered at the Tenth Anniversary Meeting of the Provincial Medical and Surgical Association, held at Exeter, Aug. 3d and 4th, 1842. By JAMES BLACK, M.D. &c. . . . 228

ART. VII.—Retrospect of the Progress of Medicine and Surgery for the year 1841-2. By Mr. E. O. SPOONER and Mr. W. SMART . . . . ib.

ART. VIII.—The Great Physician; the Connexion of Diseases and Remedies with the Truths of Revelation. By JOHN GARDNER . . . ib.

ART. IX.—On Diseases of the Bladder and Prostate Gland. With Plates. By WILLIAM COULSON . . . . . . ib.

---

### PART THIRD.—Selections from the British and Foreign Journals.

### ANATOMY AND PHYSIOLOGY.

M. BOUCHARDAT on the Composition of Fibrin . . . . 229

Revival after Freezing . . . . . . 231

M. ARAGO on the Effects of a Solar Eclipse on Animals . . . ib.

M. FOVILLE on the Decussation of Fibres at the Base of the Brain . . ib.

Dr. REMAK's Discovery of New Ganglia of Nerves . . . . 232

Prof. GROMELLI on the Erectility of the Iris . . . . 233

Mr. GULLIVER on the Pus-like Globules of the Blood . . . . 234

Mr. GULLIVER on the Structure of Fibrin, and of False Membranes. Origin of Fibre, ib.

### PATHOLOGY, PRACTICAL MEDICINE, AND THERAPEUTICS.

PAGE
M. Saint Laurent on Employing Sulphate of Quinine in Typhoid Fever . 235
M. Von Katona's Report on the Results of Inoculation in Measles . . ib.
Dr. Beer on Hairs growing on the Tongue . . . . . . ib.
L. Gmelin on the Chemical Composition of the Fluid of Ranula . . 236
Dr. Engel on the relative Frequency of Tubercles in various Organs . . ib.
Dr. Guastamacchia on a new Method of administering Quinine . . . ib.
Prof. Forget's Account of an Epidemic of Cerebro-spinal Meningitis at Strasburg. ib.
Dr. Sigg's Account of a Disease which was induced by Decayed Meat at Zurich . 238
Hogan on Quinine in Asthma . . . . . . 239

### SURGERY.

Dr. Sigmund on the Treatment of small Nævi in Children, with Acetum Lithargyri, 239
M. Bouchardat's Analysis of a Calculus removed from the Lachrymal Canal . ib.
M. Velpeau on Strabismus . . . . . . 240
M. Malgaigne's Statistical Results of the Operations in the Hospitals of Paris . ib.
M. Voillemier on the Fractures of the Lower End of the Radius . . 242
Dr. Bonnet on Extirpation of the Eye . . . . . 244
Signor Metaxa on the Removal of a whole Rib . . . . 245
Dr. Axisa on Vaccination and Revaccination . . . . . ib.
Dr. Bonnet on a New Mode of Extraction of Cataract . . . ib.
Cure of Asthenic Amaurosis by the use of Convex Spectacles . . . 246
Cases of Hepatic Abscess Explored and Punctured . . . . ib.

### MIDWIFERY, AND DISEASES OF WOMEN AND CHILDREN.

Dr. Ippolito on the Operation of Pelviotomy . . . . 247
Dr. Gilli's Account of a case in which an extraordinary number of Worms was voided by a Child . . . . . . . 248
Dr. F. von Kiwisch on Abdominal Apoplexy in New-born Children . . ib.
M. E. Boudet's History of the Epidemic of Croup which prevailed in 1840 and at the beginning of 1841, in the Hôpital des Enfans, at Paris . . 249

### MEDICAL JURISPRUDENCE AND TOXICOLOGY.

A. Devergie on the Recent Discoveries relative to processes for detecting Arsenic, 251
G. Tourdes on Cases of Asphyxia from Coal Gas . . . 252

List of Original Papers in the British Medical Journals for the Quarter ending the 20th Dec. 1842 . . . . . . 256

### Part Fourth.—Medical Intelligence.

1. Report on the Results obtained by the Use of the Microscope in the Study of Anatomy and Physiology. Part II.—On the Origin and Functions of Cells. By W. B. Carpenter, M.D. . . . (*With a Plate*) 259

2. State of Medicine in Turkey . . . . . . 282

3. Prevention of Syphilis and Cutaneous Diseases in the French Army . . ib.

4. Statistics of the Medical Profession in Norway . . . . ib.

5. Music in the Morningside (Edinburgh) Lunatic Asylum . . . ib.

Books received for Review . . . . . . 284

